# Current News

**Published:** 1901-01

**Authors:** 


### Current News.

### FOREIGN RELATIONS COMMITTEE OF THE NATIONAL ASSOCIATION OF DENTAL FACULTIES.

*To the Deans of the American Dental Colleges, Members of the National Association of Dental Faculties, and the Members of the Foreign Advisory Boards:*

GENTLEMEN,—The regulations of our National Association place the supervision of foreign educational matters which affect American colleges in the hands of the Foreign Relations Committee, and of the European Advisory Boards.

At the last annual meeting some new regulations were adopted, which limit to a certain extent the reception of foreign students, take the matter out of the exclusive hands of the Deans, and make it uniform in all the colleges by requiring the approval of the Association through its proper committee, whose decision must be reviewed at the annual meeting. We think all will comprehend the advisability of this, that the Deans may have some rule by which they may be governed and be enabled to avoid the responsibility of the acceptance of credentials in foreign tongues, with which they may not be acquainted.

We all agree upon the desirability of some uniform standard which shall apply in every case, and which will be just to all. It is quite probable that the regulations adopted at the last meeting of the National Association, which were recommended by the Foreign Relations Committee, will need amendment as we obtain further knowledge concerning foreign equivalents. The Foreign Relations Committee will each year present to the Association any new information which they may have upon the various subjects, and be prepared to make such recommendations as in their judgment will best secure the end in view. In obtaining this knowledge the Foreign Relations Committee must necessarily depend very largely upon the Advisory Boards. To these positions have been appointed the men who in the minds not only of the Foreign Relations Committee, but other well-informed members of the profession whose advice they have sought, are best qualified to perform the necessary duties. Hence we feel that we may rely upon their recommendations, and as they are so completely and honorably identified with us and are aware of the responsibility resting upon them, we do not fear that our confidence will be misplaced.

The regulations adopted at the last annual meeting of the National Association have been widely published; they have been incorporated in a pamphlet circular of information and sent to every one concerned. Hence all have had an opportunity for knowing just what was the action taken. We trust that before the next meeting of the Association all will have so fully considered the situation that they will be better enabled to act than they were when the regulations were adopted.

Since the annual meeting at Old Point Comfort, the members of the Foreign Relations Committee have carefully consulted with each other, and have unanimously arrived at the conclusion that it is not wise to attempt the enforcement of the regulations during this year, for the following reasons:

*First,* Those regulating the giving of advanced standing in our schools were not adopted until after the announcements of the colleges for the current year had already been published and distributed. It has been established by precedent that in such instances legislation should not go into effect until the next succeeding year.

*Second,* The regulations adopted at Old Point Comfort could not be promulgated until students from foreign countries had already, in some instances at least, sailed for America, and it would be an unjustifiable hardship to make them subject to new rules with which they had no opportunity to become acquainted.

*Third,* It has appeared to us advisable, before attempting to enforce them, that the new regulations should be published to the world for the benefit of all concerned, that it might be learned if in any way they work injustice, are impracticable, or are not in accordance with the laws of this or other countries.

Accordingly, the Deans who have forwarded to the Committee the papers of students have been informed they can use their discretion in the case of those from Great Britain, Germany, Austria, Switzerland, France, Holland, Belgium, Sweden, Norway, and Canada, subject always to the subsequent approval of the National Association. Even although there may be some technical lack of observation of the rules, the Foreign Relations Committee will take no cognizance of it, so long as the Deans act in good faith, always provided that the students shall obtain the approval of their papers by the Advisory Board of the country from which they come.

The above relates solely to the giving of advanced standing in our colleges on the basis of attendance on foreign schools, which may be offered as an equivalent for courses in our own institutions.

Concerning the preliminary educational and moral qualifications, and the reception of foreign students as members of the freshman class, the approval of the Advisory Board for the country from which the student comes should, in all cases, be insisted upon as provided by the regulations adopted at the annual meeting for 1899. Should a foreign student present himself without certificate, the Dean may, upon his own responsibility, receive him temporarily, on condition that within a reasonable time he shall secure such approval. If this is not done, the student shall be dismissed before the middle of the term and no credit be given him for his attendance.

This ruling of the Foreign Relations Committee may seem somewhat unnecessary, but it is deemed best that it shall be promulgated by means of this circular, that there may be no misunderstanding or misconception of the law of the Association. It is not to be understood that the Foreign Relations Committee desires to assume any authority not fully conferred upon them by the National Association, to which in every instance it is responsible. It desires to so inform the Deans and others interested that trouble may be saved, that there may be a full comprehension of the situation, and that it may not be imagined they are in any way attempting to interfere with established custom, or with the published rules of the National Association.

Yours truly,

N. C. Barrett,

*Chairman Foreign Relations Committee.*

---

### THE THIRD PAN-AMERICAN MEDICAL CONGRESS.

The Third Pan-American Medical Congress will convene in Havana, Cuba, February 5, 1901. An invitation is extended to dentists who may desire to attend and read papers before the Section on Dental and Buccal Surgery. The Committee on Transportation has made the following report on rates for the delegates at the present time: The Southeastern Passenger Association has authorized a rate of one fare for the round trip to Port Tampa, Fla., plus two dollars (exclusive of Pullman berths and meals), connecting with the Peninsula and Occidental Steamship Company at Port Tampa, which has authorized a rate of $36.50 for the round trip from Port Tampa to Havana, including meals and berths in each direction. This makes the rate through to Havana from Washington, $70.05; from Cincinnati, $68.30; from Louisville, $67.55; and correspondingly low rates from intermediate points. The Trunk Line Association has authorized excursion fares to Washington added to the fares authorized by the Southeastern Passenger Association, which includes all regular ticketing routes. The Central Traffic and Western Passenger Associations have authorized regular winter tourist rates. Delegates from these territories may find it to their interest to pay local fare to Cincinnati or Louisville, and use the authorized rates from these points as outlined above. The Ward Line steamers from New York have authorized a rate, including meals and state-room in each direction, of $60 for the round trip from New York to Havana, sailing Wednesday and Saturday; time, five days in each direction. Those who use the Ward Line to Havana, paying $60 for the round trip, can return either from Port Tampa or Miami by rail. By returning the unused portion of the ticket a rebate of $20 will be received. These tickets must be purchased from Havana over the Peninsula and Occidental Steamship Line to Port Tampa or Miami, and then by rail. No rates have been arranged so far for the New England territory. The United States Fast Mail leaving Washington over the Southern Railway at 11.15 A.M. arrives at Port Tampa, Fla., at 10.30 P.M. the next evening, making connection with the steamer leaving Port Tampa at 11 P.M., arriving at Havana the morning of the second day. Extra sleeping-cars will be run from New York over the Pennsylvania Railroad, Southern Railway, and Plant System to Port Tampa. The train leaving Cincinnati over the Queen and Crescent route at 8.30 A.M. will arrive at Port Tampa at 10.30 P. M. the following day, connecting with the same steamer. The train leaving Louisville over the Southern Railway at 7.45 A.M. connects with the Cincinnati train at Lexington, Ky., at 10.45 A.M. All these schedules unite at

Jacksonville, Fla., and go through to Port Tampa and Havana. It is suggested that the delegates from the East mobilize through Washington and those from the West through Cincinnati. Those wishing to attend will please send their own names and addresses, and of their party as well, to Eugene S. Talbot, 103 State Street, Chicago, Ill. The information will be forwarded to the Chairman of Transportation, so that sleeping-car and steamer accommodations may be reserved.

EUGENE S. TALBOT,
*Secretary Section on Dental and Buccal Surgery.*

---

### GALVESTON DENTAL SOCIETY.

GALVESTON, TEXAS, November 20, 1900.

TO OUR PROFESSIONAL BROTHERS:

Our Society met and decided to send out, for the first time perhaps in the history of dentistry, a letter asking for relief.

The late terrible disaster which swept over our city has fallen heavily upon the resident dentists; some have lost their homes, others have sustained losses which will be hard to cover, as our people are in an impoverished condition. Our practice will be a limited one for a long period.

Since we will not participate in the general relief fund, most of which has been expended in feeding and clothing the very unfortunate and in removing the wreckage, which still gives up its dead two months since the fatal night of September 8, we feel that our more fortunate brother dentists will "throw out the life line" and aid us in a small way to retrieve our losses.

The First and Second District Dental Societies of New York responded in a most generous and substantial way, and if others would follow their noble example we will soon be lifted out of our present distress. We hope to see our city in a few years what it once was, the gem of our Southland, and we a happy people but for the memory of our eight thousand relatives and friends who perished on that fatal night of September 8, 1900.

Send contributions to H. W. Lubben, President, or D. S. Killough, Secretary, 2123 Market Street, Galveston, Texas.

Respectfully,

GALVESTON DENTAL SOCIETY.

### PROGRAMME OF THE ROCHESTER DENTAL SOCIETY, SESSIONS 1900-1901.

The following essayists will read papers during the sessions of 1900-1901 before the Rochester Dental Society:

October 16, 1900, Dr. C. F. Bunbury, Esthetic Dental Prosthesis; November 13, 1900, Dr. W. W. Smith, Care and Treatment of the Dental Pulp; December 11, 1900, Professor C. H. Ward, The Welding Properties of Gold; January 8, 1901, Dr. G. Goode, Practical Sterilization of Instruments; February 12, 1901, Dr. J. W. Cowan, Pathogenic Affections of the Oral Cavity; March 12, 1901, Dr. R. H. Hofheinz, Amalgam from the Sanitary Point of View; April 9, 1901, Dr. F. Messerschmitt, Operative Prophylaxis of the Oral Cavity; May 14, 1901, Dr. J. Requa, Is Decalcified necessarily Devitalized Dentine? June 11, 1901, Dr. F. H. Lee, New Appliances and Methods; Special, Dr. J. N. Crouse, Professor Black's Experiments and the Test of Time; Special, Dr. J. Edw. Line, Tooth Decay a Race Characteristic.

---

### NEW JERSEY STATE DENTAL SOCIETY.

At the annual meeting of the New Jersey State Dental Society the following officers were elected for the ensuing year: President, Dr. F. Edsall Riley, Newark; Vice-President, Dr. Wm. L. Fish, Newark; Treasurer, Dr. Henry A. Hull, New Brunswick; Secretary, Dr. Charles A. Meeker, 29 Fulton Street, Newark; Assistant Secretary, Dr. H. S. Sutphen, 24 East Kinney Street, Newark.

*Executive Committee.*—Dr. H. S. Sutphen, Newark; Dr. Oscar Adelberg, Elizabeth; Dr. F. L. Hindle, New Brunswick; Dr. W. H. Pruden, Paterson.

*Membership Committee.*—Dr. G. M. Holden, Chairman, Hackettstown; Dr. J. L. Crater, Orange; Dr. W. W. Hawke, Flemington; Dr. A. Irwin, Camden; Dr. W. W. Woolsey, Elizabeth.

C. A. Meeker,
*Secretary.*

